# Supplementary material for: Translating genomics into practice for real-time surveillance and response to carbapenemase-producing Enterobacteriaceae: evidence from a complex multi-institutional KPC outbreak
Source: PeerJ. 2018 Jan 3;6:e4210. doi: 10.7717/peerj.4210 (PMC5756455; doi:10.7717/peerj.4210)
Supplement: Supplemental Information 1 [file peerj-06-4210-s001.pdf]

Translating genomics into practice for real-time surveillance and response to carbapenemase-producing Enterobacteriaceae: evidence from a complex multi-institutional KPC outbreak

Kwong JC [1,2,3], Lane C [2,4], Romanes F [4], Gonçalves da Silva A [1,2], Easton M[4], Cronin K [5], Waters MJ [5], Tomita T [2], Stevens K [2], Schultz M [1,2], Baines S [1,2], Sherry NL [2,3], Carter G [1], Mu A [1], Sait M [2], Ballard S [1,2], Seemann T [1,2,6], Stinear TP [1] & Howden BP [1,2,3].

[1] Doherty Applied Microbial Genomics, and [2] Microbiological Diagnostic Unit Public Health Laboratory, Department of Microbiology and Immunology, The University of Melbourne at The Peter Doherty Institute for Infection & Immunity, Melbourne, Australia
[3] Department of Infectious Diseases, Austin Health, Heidelberg, Australia
[4] Communicable Diseases Branch, Department of Health & Human Services, Victoria State Government, Melbourne, Australia
[5] Department of Microbiology, St Vincent's Hospital Melbourne, Fitzroy, Australia
[6] Melbourne Bioinformatics, Carlton, Australia

Supplementary Appendix

Table S1: List of primers used for detection of carbapenemase genes. Carbapenemase genes were detected in suspected CPE isolates with a multiplex PCR using the primers listed. Reported genotypes were confirmed by Sanger sequencing.

Gene/Target	Primer	Primer sequence (5' -3')	PCR product size (bp)	Reference
bla_{KPC}	KpcF	ATG TCA CTG TAT CGC CGT C	845	[5]
	KpcR	TTA CTG CCC GTT GAC GCC-3'		
$bla_{OXA\text{-}48\text{-}like}$	OXA-48F	TTG GTG GCA TCG ATT ATC GG	743	[6]
	OXA-48R	GAG CAC TTC TTT TGT GAT GGC		
bla_{IMP}	IMP-A	GAA GGY GTT TAT GTT CAT AC	587	[7]
	IMP-B	GTA MGT TTC AAG AGT GAT GC		
bla_{VIM}	VIM2004-A	GTT TGG TCG CAT ATC GCA AC	382	[7]
	VIM2004-B	AAT GCG CAG CAC CAG GAT AG		
bla_{NDM}	NDM-F	GGG CAG TCG CTT CCA ACG GT	475	[8]
	NDM-R	GTA GTG CTC AGT GTC GGC AT		
IMP sequencing:	5'CS	GGC ATC CAA GCA GCA AG	793	[7]
5'CS-IMP-B	IMP-B	GTA MGT TTC AAG AGT GAT GC		
		Same as bla_{IMP} PCR primer		
VIM 1 amplification	Vim-1F	TTA TGG AGC AGC AAC GAT GT	920	[9]
& sequencing	Vim-1R	CAA AAG TCC CGC TCC AAC GA		
VIM-2 amplification	Vim-2F	AAA GTT ATG CCG CAC TCA CC	865	
& sequencing	Vim-2R	TGC AAC TTC ATG TTA TGC CG		
VIM-2 sequencing	Vim-2sF	TCG ACG GTG ATG CGT ACG TT		
	Vim-2sR	TTG ATG TCC TTC GGG CGG CT		
NDM sequencing	NDMLF	ATG GAA TTG CCC AAT ATT ATG CAC	813	[10]
	NDMLR	TCA GCG CAG CTT GTC GGC		
bla_{MOX}	MOXF	GCT GCT CAA GGA GCA CAG GAT	520	[11]
	MOXR	CAC ATT GAC ATA GGT GTG GTG C		
bla_{CIT}	CITF	TGG CCA GAA CTG ACA GGC AAA	462	
	CITR	TTT CTC CTG AAC GTG GCT GGC		
bla_{DHA}	DHAF	AAC TTT CAC AGG TGT GCT GGG T	405	
	DHAR	CCG TAC GCA TAC TGG CTT TGC		
bla_{ACC}	ACCF	AAC AGC CTC AGC AGC CGG TTA	346	
	ACCR	TTC GCC GCA ATC ATC CCT AGC		
bla_{EBC}	EBCF	TCG GTA AAG CCG ATG TTG CGG	302	
	EBCR	CTT CCA CTG CGG CTG CCA GTT		
bla_{FOX}	FOXF	AAC ATG GGG TAT CAG GGA GAT G	190	
	FOXR	CAA AGC GCG TAA CCG GAT TGG'		
$bla_{OXA\text{-}23\text{-}like}$	OXA-23F	GAT CGG ATT GGA GAA CCA GA	5001	[12]
	OXA-23R	ATT TCT GAC CGC ATT TCC AT		
$bla_{OXA\text{-}24\text{-}like}$	OXA-24F	GGT TAG TTG GCC CCC TTA AA	246	
	OXA-24R	AGT TGA GCG AAA AGG GGA TT		
$bla_{OXA\text{-}51\text{-}like}$	OXA-51F	TAA TGC TTT GATCGG CCT TG	353	
	OXA-51R	TGG ATT GCA CTT CAT CTT GG		
$bla_{OXA\text{-}58\text{-}like}$	OXA-58F	AAG TAT TGG GGC TTG TGC TG	599	
	OXA-58R	CCC CTC TGC GCT CTA CAT AC		
IS*Aba1*	Int1F	CAG TGG ACA TAA GCC TGT TC	160	[13]
	Int1R	CCC GAG GCA TAG ACT GTA		

Table S2: List of NCBI *K. pneumoniae* genomes used for initial comparison (Figure S2).

KP_1084	KP_CHS109	KP_CHS235	KP_MGH_19
KP_1158	KP_CHS110	KP_CHS236	KP_MGH_20
KP_32192	KP_CHS112	KP_CHS82	KP_MGH_21
KP_342	KP_CHS114	KP_CHS85	KP_MGH_30
KP_34618	KP_CHS115	KP_CHS89	KP_MGH_31
KP_6234	KP_CHS116	KP_CHS90	KP_MGH_32
KP_ATCC_13883	KP_CHS118	KP_CHS91	KP_MGH_35
KP_ATCC_BAA-2146	KP_CHS121	KP_CHS92	KP_MGH_36
KP_BAMC_07-18	KP_CHS122	KP_CHS96	KP_MGH_39
KP_BIDMC85	KP_CHS125	KP_CHS97	KP_MGH_40
KP_BIDMC86	KP_CHS126	KP_CHS98	KP_MGH_43
KP_BIDMC88	KP_CHS127	KP_CHS99	KP_MGH_44
KP_BIDMC89	KP_CHS132	KP_CHS_07	KP_MGH_45
KP_BIDMC91	KP_CHS133	KP_CHS_11	KP_MGH_47
KP_BIDMC95	KP_CHS134	KP_CHS_12	KP_MGH_48
KP_BIDMC96	KP_CHS137	KP_CHS_17	KP_MGH_64
KP_BIDMC_1	KP_CHS139	KP_CHS_30	KP_MGH_66
KP_BIDMC_12C	KP_CHS140	KP_CHS_34	KP_MGH_68
KP_BIDMC_16	KP_CHS144	KP_CHS_48	KP_MGH_73
KP_BIDMC_18A	KP_CHS147	KP_CHS_49	KP_MGH_78578
KP_BIDMC_18C	KP_CHS148	KP_CHS_50	KP_NJST258_1
KP_BIDMC_22	KP_CHS149	KP_CHS_54	KP_NJST258_2
KP_BIDMC_23	KP_CHS150	KP_CHS_55	KP_NTUH-K2044
KP_BIDMC_24	KP_CHS151	KP_CHS_59	KP_PMK1
KP_BIDMC_25	KP_CHS154	KP_CHS_61	KP_PittNDM01
KP_BIDMC_2A	KP_CHS156	KP_CHS_63	KP_SA1
KP_BIDMC_31	KP_CHS162	KP_CHS_70	KP_T69
KP_BIDMC_34	KP_CHS164	KP_CHS_71	KP_UCI70
KP_BIDMC_35	KP_CHS165	KP_CHS_72	KP_UCI76
KP_BIDMC_36	KP_CHS167	KP_CHS_73	KP_UCI81
KP_BIDMC_42a	KP_CHS168	KP_CHS_76	KP_UCI82
KP_BIDMC_42b	KP_CHS169	KP_CHS_80	KP_UCI91
KP_BIDMC_46a	KP_CHS174	KP_Ecl8	KP_UCI96
KP_BIDMC_46b	KP_CHS179	KP_FDAARGOS_84	KP_UCICRE_1
KP_BIDMC_47	KP_CHS185	KP_HK787	KP_UCICRE_10
KP_BIDMC_48	KP_CHS186	KP_HS11286	KP_UCICRE_13
KP_BIDMC_5	KP_CHS191	KP_JM45	KP_UCICRE_2
KP_BIDMC_51	KP_CHS194	KP_KCTC_2242	KP_UCICRE_4
KP_BIDMC_52	KP_CHS200	KP_KP5-1	KP_UCICRE_6
KP_BIDMC_53	KP_CHS201	KP_KPNIH1	KP_UCICRE_7
KP_BIDMC_55	KP_CHS202	KP_KPNIH10	KP_UCI_17
KP_BIDMC_60	KP_CHS205	KP_KPNIH24	KP_UCI_18
KP_BIDMC_61	KP_CHS207	KP_KPNIH27	KP_UCI_19
KP_BIDMC_7B	KP_CHS208	KP_KPR0928	KP_UCI_21
KP_BJ1-GA	KP_CHS210	KP_Kp13	KP_UCI_25
KP_BWH53	KP_CHS211	KP_Kp52	KP_UCI_26
KP_BWH62	KP_CHS214	KP_LCT-KP214	KP_UCI_34
KP_BWH_2	KP_CHS215	KP_MGH101	KP_UCI_41
KP_BWH_22	KP_CHS216	KP_MGH102	KP_UCI_42
KP_BWH_28	KP_CHS218	KP_MGH112	KP_UCI_43
KP_BWH_30	KP_CHS219	KP_MGH115	KP_UCI_56
KP_BWH_41	KP_CHS221	KP_MGH116	KP_UCI_59
KP_BWH_46	KP_CHS223	KP_MGH117	KP_UCI_62
KP_CAV1344	KP_CHS224	KP_MGH124	KP_WGLW1
KP_CAV1392	KP_CHS225	KP_MGH82	KP_WGLW2
KP_CAV1596	KP_CHS226	KP_MGH84	KP_WGLW3
KP_CG43	KP_CHS228	KP_MGH89	KP_XH209
KP_CHS101	KP_CHS229	KP_MGH90	KP_blaNDM-1
KP_CHS102	KP_CHS230	KP_MGH94	KP_rh_SB3432
KP_CHS104	KP_CHS231	KP_MGH96	KP_yzusk-4
KP_CHS105	KP_CHS232	KP_MGH_18	

Table S3: List of NCBI accessions for CC258 *K. pneumoniae* included for comparison (Figure S3).

SAMN01057606	SRR1166990	SRR1561228	SRR1561301
SAMN01057607	SRR1166991	SRR1561231	SRR1561302
SAMN01057608	SRR1166992	SRR1561232	SRR1561303
SAMN01057609	SRR1166993	SRR1561233	SRR1561305
SAMN01057611	SRR1166994	SRR1561234	SRR1561306
SAMN01057612	SRR1166995	SRR1561235	SRR1561308
SAMN01057613	SRR1166996	SRR1561237	SRR1561309
SAMN01057614	SRR1166997	SRR1561239	SRR1561311
SAMN01057620	SRR1166998	SRR1561240	SRR1561312
SAMN01057621	SRR1166999	SRR1561241	SRR1561313
SAMN01057635	SRR1167000	SRR1561242	SRR1561315
SAMN01057638	SRR1167001	SRR1561243	SRR1561316
SAMN01057640	SRR1167002	SRR1561244	SRR1561317
SAMN01057641	SRR1167003	SRR1561245	SRR1561318
SAMN01057646	SRR1167004	SRR1561246	SRR1561320
SAMN01057650	SRR1167005	SRR1561247	SRR1561321
SAMN01057652	SRR1167006	SRR1561248	SRR1561322
SAMN01057657	SRR1167007	SRR1561249	SRR1561327
SAMN01057658	SRR1167008	SRR1561251	SRR1561328
SAMN01057711	SRR1167009	SRR1561252	SRR1561329
SAMN02786855	SRR1167010	SRR1561253	SRR1561330
SRR1166945	SRR1167011	SRR1561254	SRR1561331
SRR1166946	SRR1167012	SRR1561255	SRR1561332
SRR1166947	SRR1167013	SRR1561256	SRR1561333
SRR1166948	SRR1167014	SRR1561257	SRR1561334
SRR1166949	SRR1167015	SRR1561258	SRR1561336
SRR1166950	SRR1167016	SRR1561259	SRR1561339
SRR1166951	SRR1167017	SRR1561260	SRR1561341
SRR1166952	SRR1167018	SRR1561261	SRR1561342
SRR1166953	SRR1167019	SRR1561262	SRR1561343
SRR1166954	SRR1167020	SRR1561263	SRR1561344
SRR1166955	SRR1167021	SRR1561264	SRR1561345
SRR1166956	SRR1167022	SRR1561265	SRR1561346
SRR1166957	SRR1167023	SRR1561266	SRR1561347
SRR1166958	SRR1167024	SRR1561267	SRR1561349
SRR1166959	SRR1167025	SRR1561268	SRR1561350
SRR1166960	SRR1167026	SRR1561269	SRR1561351
SRR1166961	SRR1167027	SRR1561270	SRR1561352
SRR1166962	SRR1167028	SRR1561271	SRR1561353
SRR1166963	SRR1561198	SRR1561272	SRR1561354
SRR1166964	SRR1561199	SRR1561273	SRR1561355
SRR1166965	SRR1561200	SRR1561274	SRR1561356
SRR1166966	SRR1561201	SRR1561275	SRR1561360
SRR1166967	SRR1561202	SRR1561276	SRR1561363
SRR1166968	SRR1561203	SRR1561277	SRR1561364
SRR1166969	SRR1561204	SRR1561278	SRR1582856
SRR1166970	SRR1561205	SRR1561279	SRR1582857
SRR1166971	SRR1561206	SRR1561280	SRR1582862
SRR1166972	SRR1561207	SRR1561281	SRR1582863
SRR1166973	SRR1561208	SRR1561282	SRR1582864
SRR1166974	SRR1561209	SRR1561283	SRR1582865
SRR1166975	SRR1561210	SRR1561284	SRR1582866
SRR1166976	SRR1561211	SRR1561285	SRR1582867
SRR1166977	SRR1561215	SRR1561286	SRR1582868
SRR1166978	SRR1561216	SRR1561287	SRR1582870
SRR1166979	SRR1561217	SRR1561288	SRR1582871
SRR1166980	SRR1561218	SRR1561289	SRR2033747
SRR1166981	SRR1561219	SRR1561290	SRR2033748
SRR1166982	SRR1561220	SRR1561291	SRR2033756
SRR1166983	SRR1561221	SRR1561292	SRR2033781
SRR1166984	SRR1561222	SRR1561293	SRR2033783
SRR1166985	SRR1561223	SRR1561294	SRR2033784
SRR1166986	SRR1561224	SRR1561295	SRR2033785
SRR1166987	SRR1561225	SRR1561296	SRR2033787
SRR1166988	SRR1561226	SRR1561297	
SRR1166989	SRR1561227	SRR1561298	

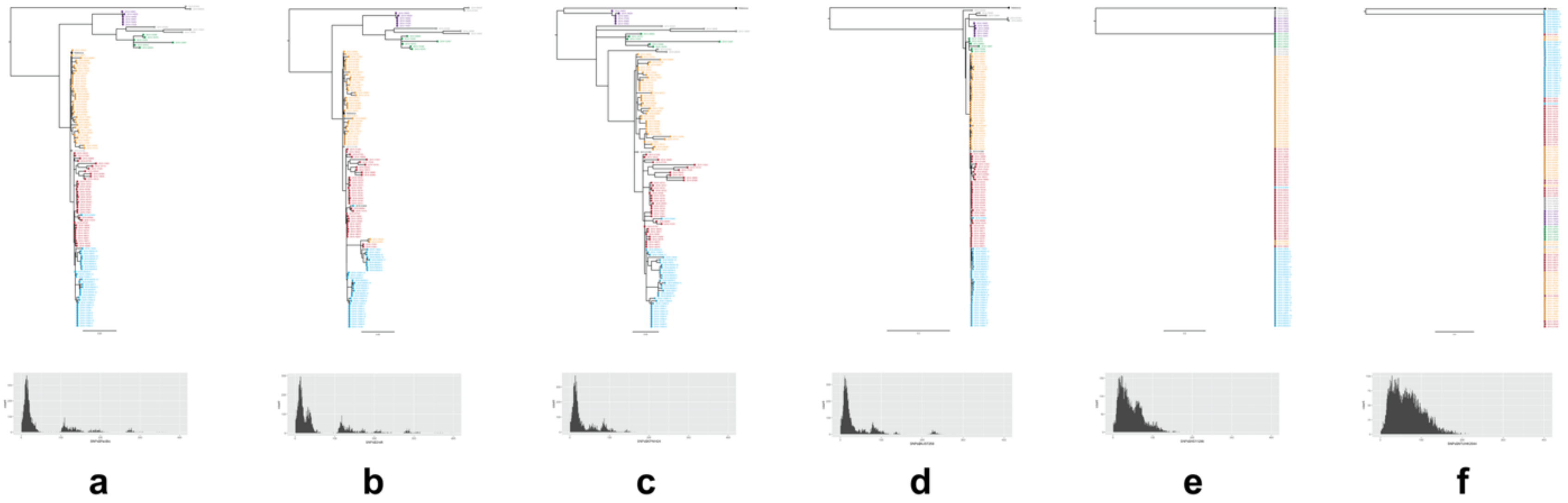

Figure S1a: Effect of different reference genome selection on phylogenetic structure:

a) Closed genome assembly of PacBio-sequenced local *K. pneumoniae* isolate
b) Draft genome assembly of Illumina-sequenced local *K. pneumoniae* isolate
c) KPNIH24 – GenBank genome, same clade I (based on capsular locus type) CC258 *K. pneumoniae*
d) NJST258_1 – GenBank genome, clade II CC258 *K. pneumoniae*
e) HS11286 – GenBank genome, ST11 *K. pneumoniae* (ancestral clone to ST258; shares six MLST alleles)
f) NTUH-K2044 – GenBank genome, ST23 *K. pneumoniae*

Each phylogenetic tree represents the same group of isolates, with each tree inferred from an alignment of core-genome SNPs using each of the respective reference genomes using identical methods. Tree tips have been coloured as follows:
Genomic cluster A (green), cluster B1 (orange), cluster B2 (red), cluster C (purple); within-host diversity isolates from patient 70 (blue).

Histograms below show distribution of pairwise SNP distances (as in Figure S5a) using each respective reference genome.

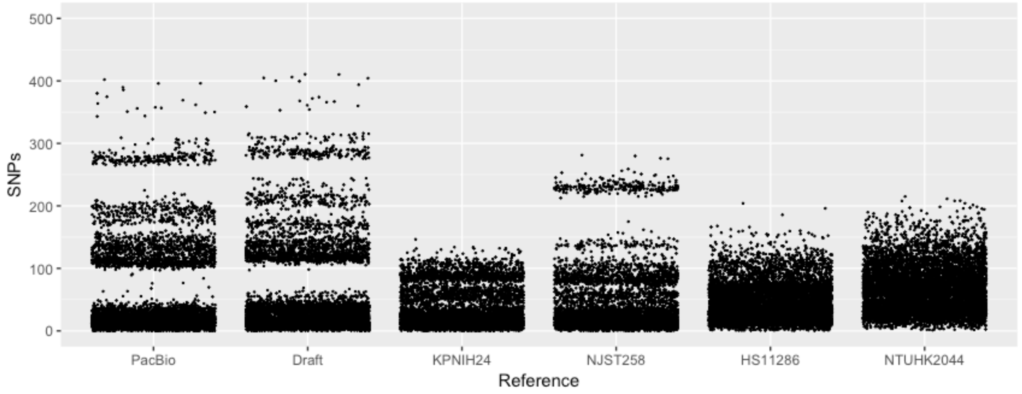

Figure S1b: Scatterplots of the pairwise SNP distances corresponding to each of the reference genomes used.

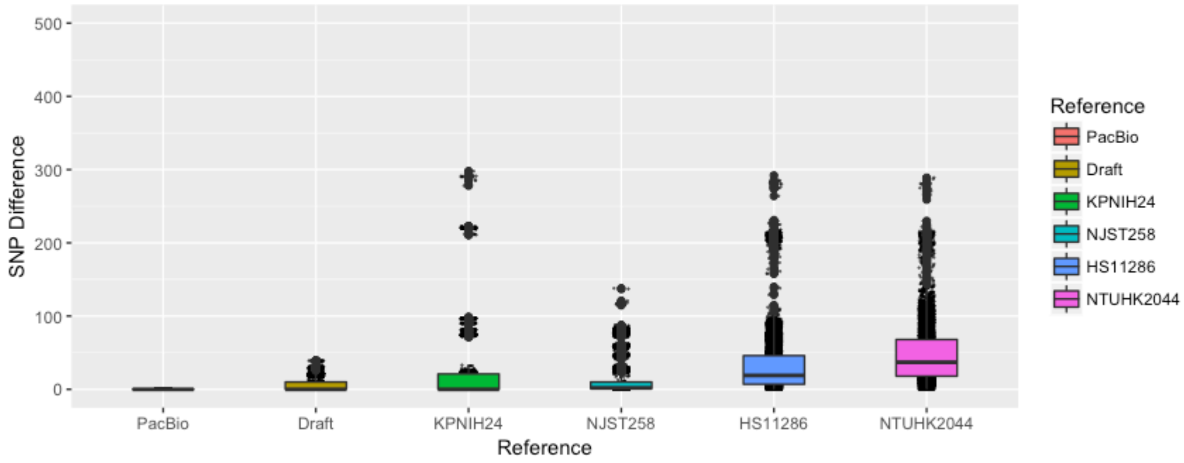

Figure S1c: Difference in reported pairwise SNP distance for each pair of isolates using different reference genomes compared to using the PacBio assembled internal reference genome. Boxes show median difference and interquartile ranges.

Table S4: KPC-producing Enterobacteriaceae isolates until 31 December 2015

**Species (and sequence type [ST])**	
Klebsiella pneumoniae	
ST16	1
ST39	1
ST147	2
ST258	73
ST1048	1
Novel ST [a]	1
Klebsiella oxytoca	
ST146	1
Citrobacter farmeri	5
Citrobacter freundii	1
**Specimen source**	
Blood	10 (12%)
Urine	42 (49%)
Sputum	4 (5%)
Aspirate fluid	4 (5%)
Wound swab	2 (2%)
Tissue	2 (2%)
Rectal swab / Faeces	20 (23%)
Other [b]	2 (2%)
**Antibiotic resistance [c]**	
K. pneumoniae (n = 58)	
Meropenem	57 (98%) [d]
Amikacin	55 (95%)
Gentamicin	44 (76%)
Ciprofloxacin	57 (98%)
Trimethoprim-Sulfamethoxazole	46 (79%)
C. farmeri (n = 4)	
Meropenem	4 (100%)
Amikacin	4 (100%)
Gentamicin	4 (100%)
Ciprofloxacin	3 (75%)
Trimethoprim-Sulfamethoxazole	4 (100%)
**Carbapenemase gene present (by WGS)**	
KPC-2	83 (97%) [d]
KPC-3	2 (2%)

[a] Novel *tonB* allele and allele combination (non CC258)
[b] Other = pharyngeal swab (n = 1), unknown (n = 1)
[c] Resistance or intermediate resistance present by Clinical and Laboratory Standards Institute (CLSI) breakpoints
[d] One ST258 isolate initially tested resistant to meropenem, but was susceptible on repeat testing and found to lack *bla*KPC-2

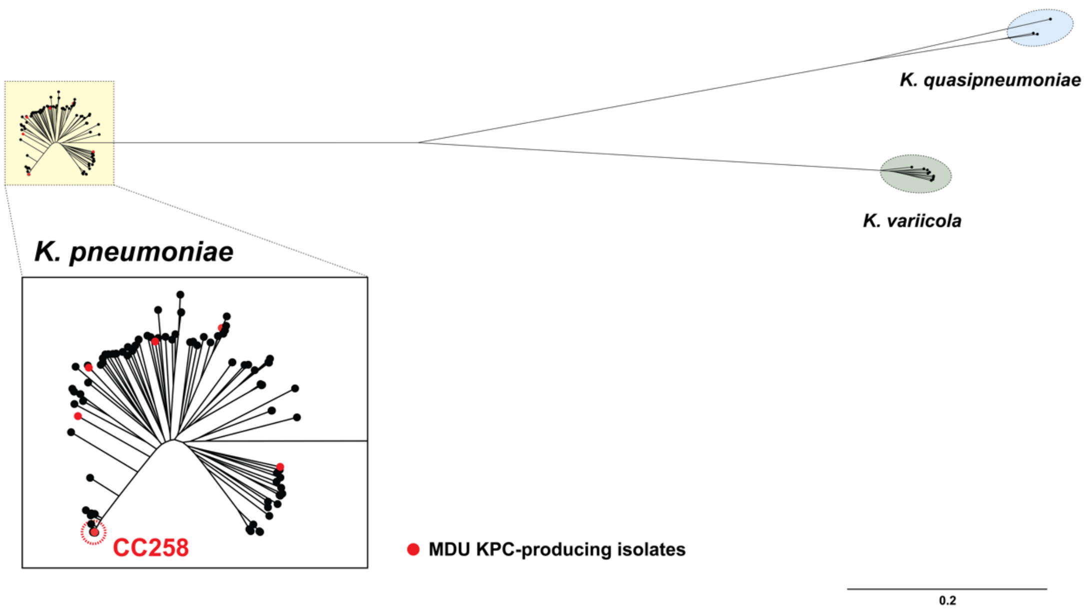

Figure S2: Approximate maximum likelihood tree showing comparison between KPC-producing *K. pneumoniae* isolates referred to MDU and publicly available *K. pneumoniae* genomes in GenBank. The tree was inferred from an alignment of core genome SNPs using *FastTree* v2.1.8 [2]. Genomes of local isolates referred to MDU are shown in red, including 29 CC258 isolates (circled).

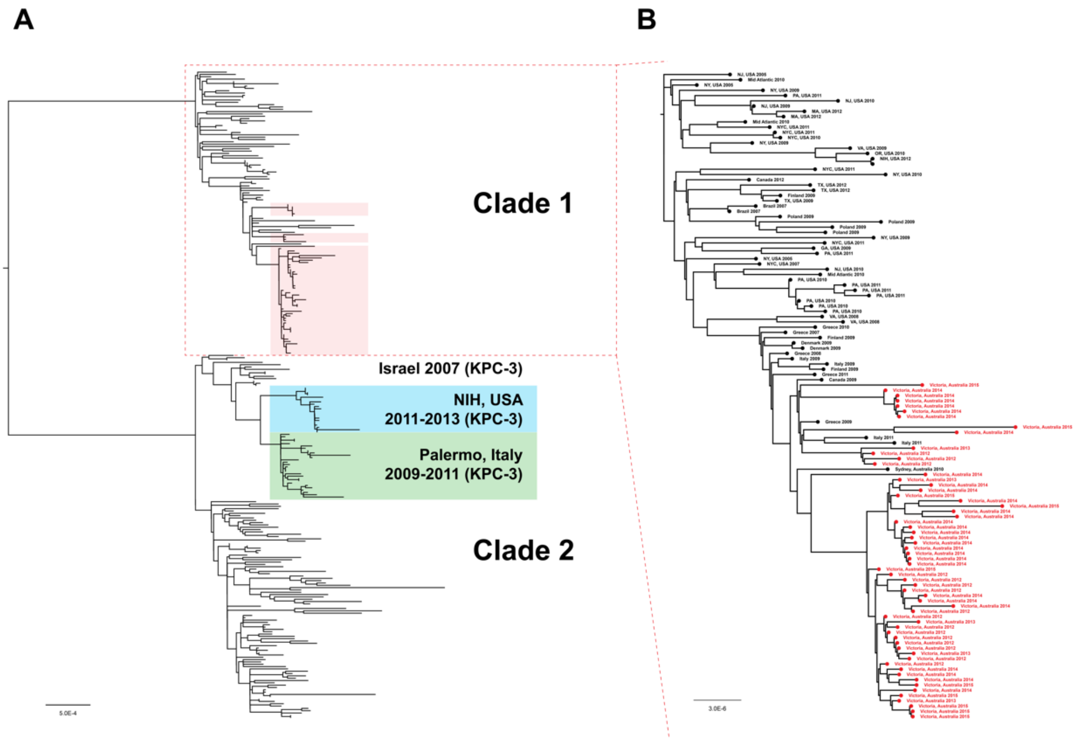

Figure S3: A) Approximate maximum likelihood tree showing phylogenetic context of KPC-producing CC258 *K. pneumoniae* referred to MDU (shaded in pink) in relation to other publicly available CC258 *K. pneumoniae* genomes in GenBank. B) expanded figure for Clade 1 CC258 *K. pneumoniae*.

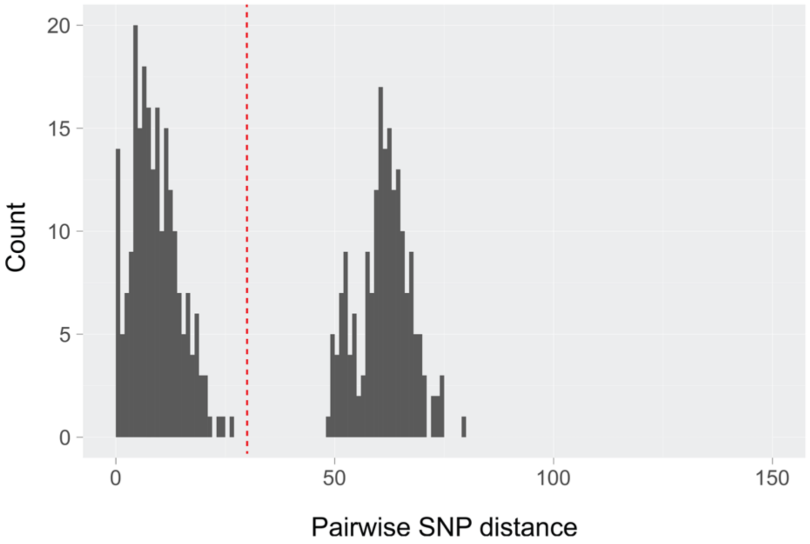

a)

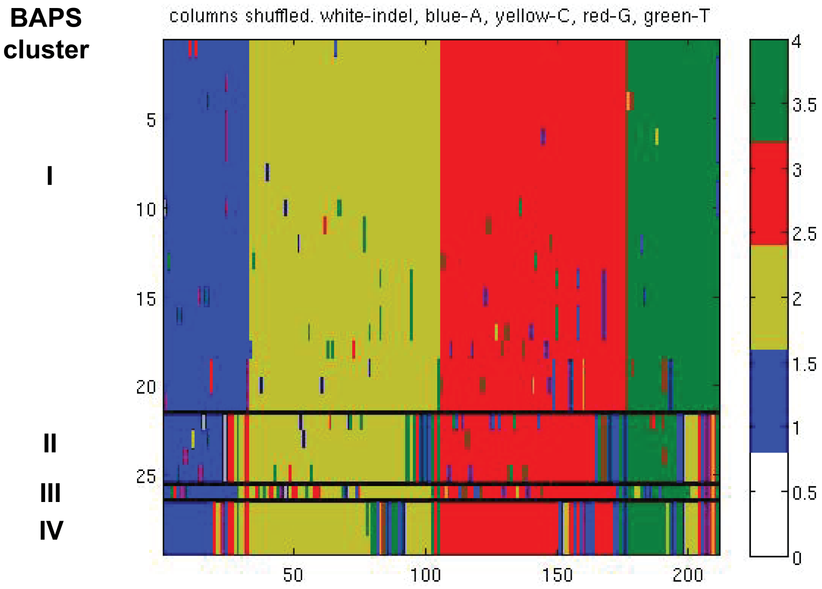

b)

Figure S4: Support for defining genomic clusters. **a)** Histogram of pairwise SNP distances between each pair of isolates in the initial genomic analysis showing a binomial distribution. Assuming the pairwise SNP distance between isolates in the same cluster is lower than the SNP distance between two isolates in different clusters, a threshold distance of 30 SNPs (red dashed line) applied to the initial phylogeny shown in Figure 3 identified three distinct clusters (labelled A, B, C). **b)** Bayesian Analysis of Population Structure (BAPS) analysis using hierarchical BAPS [3], with clustering performed iteratively to 8 levels in the hierarchy, and the maximum number of clusters prior set at 10. Four BAPS clusters were identified, corresponding to the 3 major clades (A, B, C) shown in Figure 3 in addition to a single outlier.

Table S5: Demographic and clinical characteristics, KPC-2 postive CC258 *K. pneumoniae* colonised patients, Victoria, categorised by genomic cluster assignment.

	Cluster				Unclustered (n=4)	Total (n=57)
	**A (n=4)**	**B1 (n=28)**	**B2 (n=16)**	**C (n=5)**		
Age, yrs (median, range)	76 (74-83)	67 (20-90)	81 (52-95)	74 (58-78)	82 (73-88)	74 (20-95)
Male (n, %)	1 (25)	14 (50)	11 (69)	3 (60)	4 (100)	33 (58)
Year first isolate identified	2012	2012	2013	2014	2014	
**Colonised patients**	**0 (0)**	**4 (7)**	**9 (56)**	**2 (40)**	**3 (75)**	**18 (32)**
**Infected patients (n, %)**	**4 (100)**	**22 (79)**[b]	**5 (31)**[b]	**3 (60)**	**1 (25)**	**35 (61)**
Site of infection						
Urinary tract	4 (100)	13 (46)	4 (14)	2 (40)	1 (25)	24 (42)
Intrabdominal	-	5 (18)	-	-	-	5 (9)
Pulmonary & pleural	-	3 (11)	1 (4)	-	-	4 (7)
Intravascular line-related	-	1 (4)	-	-	-	1 (2)
Skin/soft tissue	-	-	-	1 (4)	-	1 (2)
Sepsis syndrome	1 (25)	15 (54)	3 (19)	2 (40)	1 (25)	22 (39)
**Comorbidities**						
Kidney/renal disease	2 (50)	15 (54)	12 (75)	1 (20)	1 (25)	31 (54)
Type 2 diabetes	2 (50)	7 (25)	8 (50)	1 (20)	-	18 (32)
Liver disease	1 (25)	10 (36)	1 (6)	-	-	12 (21)
Prior chemo/radiotherapy	-	7 (25)	16 (13)	-	1 (25)	10 (18)
Antibiotic therapy > 1 month	2 (50)	8 (29)	5 (31)	3 (60)	1 (25)	19 (33)
**Hospitalisation in prior 12 months**	**4 (100)**	**28 (100)**	**16 (100)**	**5 (100)**	**4 (100)**	**57 (100)**
Healthcare Facility I	3 (75)	-	-	-	-	3 (5)
Healthcare Facility F	-	23 (82)	16 (100)	-	-	39 (68)
Healthcare Facility A	-	-	-	4 (80)	-	4 (7)
**Overseas travel in prior 12 months**[c]	**-**	**1 (4)**	**1 (6)**	**2 (40)**	**4 (100)**	**8 (14)**
Country of travel[a]						
Greece	-	-	-	1 (20)	4 (100)	5 (9)
New Zealand	-	1 (4)	-	-	-	1 (2)
Vietnam	-	-	1 (6)	1 (20)	-	2 (4)
Thailand	-	-	-	1 (20)	-	1 (2)
Hong Kong	-	-	-	1 (20)	-	1 (2)
Overseas hospitalisation	-	-	-	2 (40)	4 (100)	6 (11)

[a] Sum may exceed total where patients report multiple travel destinations
[b] Infection/colonisation status unknown for some patients
[c] Travel history unknown for 11 patients

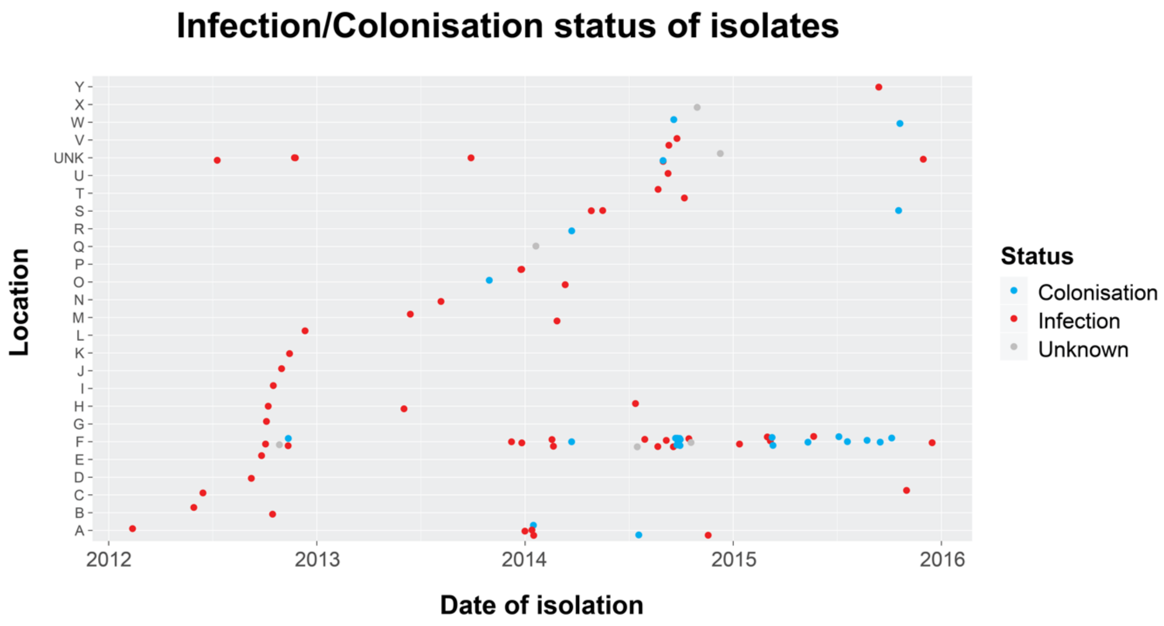

Figure S5: Infection/colonisation status of KPC-producing isolates, by date and location of isolation. Data were plotted in R using ggplot2.

Supplementary epidemiological data

A total of 113 requests for patient hospitalisation data from forty-one separate healthcare facilities were made, 105 (93%) of which were able to be filled. Data were gathered on 2165 healthcare presentations. Interviews with the general practitioner, or alternate practice staff member, was completed for 48/57 (84%) of patients. Two patients had no record of a general practitioner. The remaining seven general practitioners were unable to be contacted. Patient or next of kin interviews were completed for 46/57 (81%) of patients, 11 of which were conducted with the patient and 34 with one or more next of kin. Of the remaining 11 patients, four were unable to be contacted, two refused to give information despite successful contact, three were deemed not for contact by their general practitioner and two were deceased or experiencing severe communication difficulties and without identifiable next of kin living in Australia.

Data on patient sex, overseas travel and recent hospitalisation are shown in Supplementary Table S5. Country of birth was known for 51/57 (89%) of patients. Of those, 20 (39%) were born in Australia, 9 (16%) were born in Italy, and 8 (14%) in Greece. Year of arrival in Australia was known for 20 of the 31 patients born overseas and ranged from 1950 to 2000 (mean 1965). Only one patient, born in New Zealand, had migrated to Australia since the emergence of KPC in 1996. Of those that had travelled, the most common overseas destinations were Greece (n=6) and Italy (n=6), also corresponding with the most common overseas countries of birth. Of the eight patients that had travelled in the 12 months prior to isolation, six had been hospitalised overseas, five in Greece and one in both Vietnam and Thailand. All patients with isolates that did not fall into one of the major local phylogenetic clades reported overseas hospitalisation in the 12 months prior.

Based on clinical record review, colonisation (in the absence of recognised infection) was the probable manifestation for 18 of the 57 patients (32%) with a ST258 KPC-2 *K. pneumoniae* isolate in Victoria. In a further 35 (61%) patients, clinical notes either directly stated *K. pneumoniae* as the cause of infection, or detailed an illness clinically compatible with the site of specimen at the time of collection. Of those with symptomatic infection, the most common site of infection was urinary tract (n=24, 42%), followed by intra-abdominal (n=5, 9%) and pulmonary and pleural infection (n=4, 7%), with skin and soft tissue and intra-vascular line related infection each occurring in one patient. Twenty-two patients (39%) had a sepsis syndrome. The clinical significance of the KPC-producing isolate in the remaining four patients was unknown. Directed antimicrobial treatment for infection due to a KPC-producing organism was commenced in 25 (71%) of the 35 symptomatically infected patients. One patient with a skin and soft tissue infection was treated surgically. In two patients, medical notes state that directed treatment was withheld due to palliative intent. The proportion of cases with symptomatic infection appeared to vary between the genomic clusters, and may be

reflective of screening practices in different facilities, though numbers were small and not significantly different (p = 0.208, Fisher's Exact Test).

Data were collected on comorbidities, though no significant associations were identified. Renal disease was the most commonly reported comorbidity (54%), though may have just reflected the age of the study population. At least 30 patients (53%) had an indwelling medical device (excluding peripherally inserted intravenous canulas) at the time of first isolation, including urinary catheters, central venous catheters, and surgical drains – known risk factors for KPC acquisition [1]. The most common indwelling medical device was a urinary catheter in 13 of the 57 patients (23%), followed by eight patients found to have ureteric, biliary or renal stents; however six of these eight patients (75%) had isolates from genomic cluster B1 and were thought to reflect admission to a location of transmission, rather than risk of KPC acquisition itself.

Information on residence at aged care facilities was available for 53 of the 57 patients, of which 12 (23%) are believed to have resided in an aged care facility at any time. Dates of residence were known for 11 of the 12 patients, with only three residing in the aged care facility prior to first KPC-2 producing *K. pneumoniae* detection. No aged care facility was reported as a location of residence for more than one patient.

Amongst those who were inpatients on the day of collection of their first KPC-2 producing *K. pneumoniae* specimen, and for whom data was available, the median time from admission to detection was seven days (range 0-93 days). After excluding patients where specimen collection was within 48 hours of admission, the median time from admission to isolation was 17 days (range 3-93, n=33). Median number of inpatient days in Australian hospitals, in the 12 months prior to admission, for all patients was 62 days (range 1-182). Four patients had fewer than ten Australian inpatient days in the 12 months prior to first KPC-2 producing *K. pneumoniae* isolation. Of these four patients, three had been hospitalised overseas in the 12 months prior, for which exact dates and duration of hospitalisation is not known. The relationships between genomic cluster and the healthcare facilities to which patients presented in the 12 months prior to their first known KPC-2 producing *K. pneumoniae* detection are apparent in the data (Table S5). Significant differences exist in the proportion presenting to the selected healthcare services amongst genomic cluster A (Cochrane's Q test, $p<0.001$), genomic cluster B ($p<0.001$), genomic cluster C ($p<0.001$) and genomic cluster D ($p<0.001$). No such differences existed in isolates classified as "other" (Cochrane's Q test, $p=0.273$).

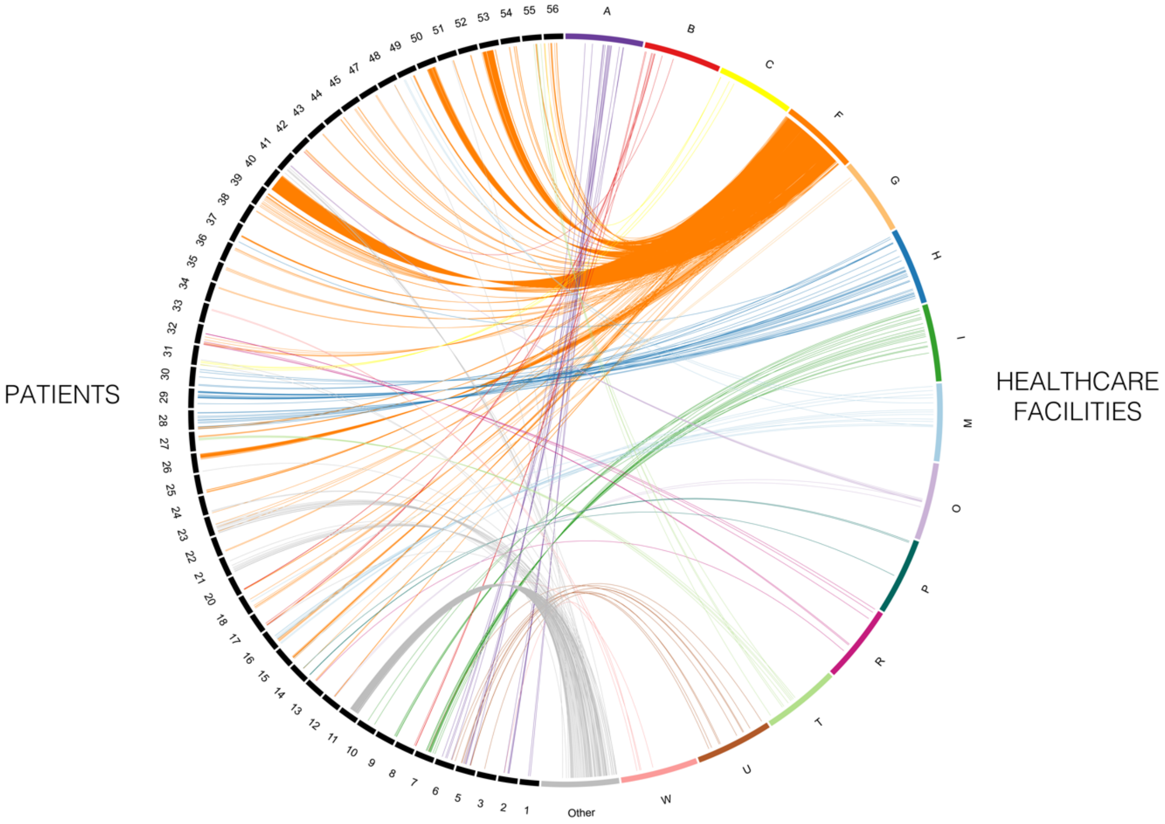

Figure S6: Unordered patient admission data from the investigation, showing patient movements among healthcare facilities. Coloured bands linking patients (left) to healthcare facilities (right) represent separate healthcare admissions, with the width of the band corresponding to the duration of the admission. The figure was drawn using Circos v0.67.

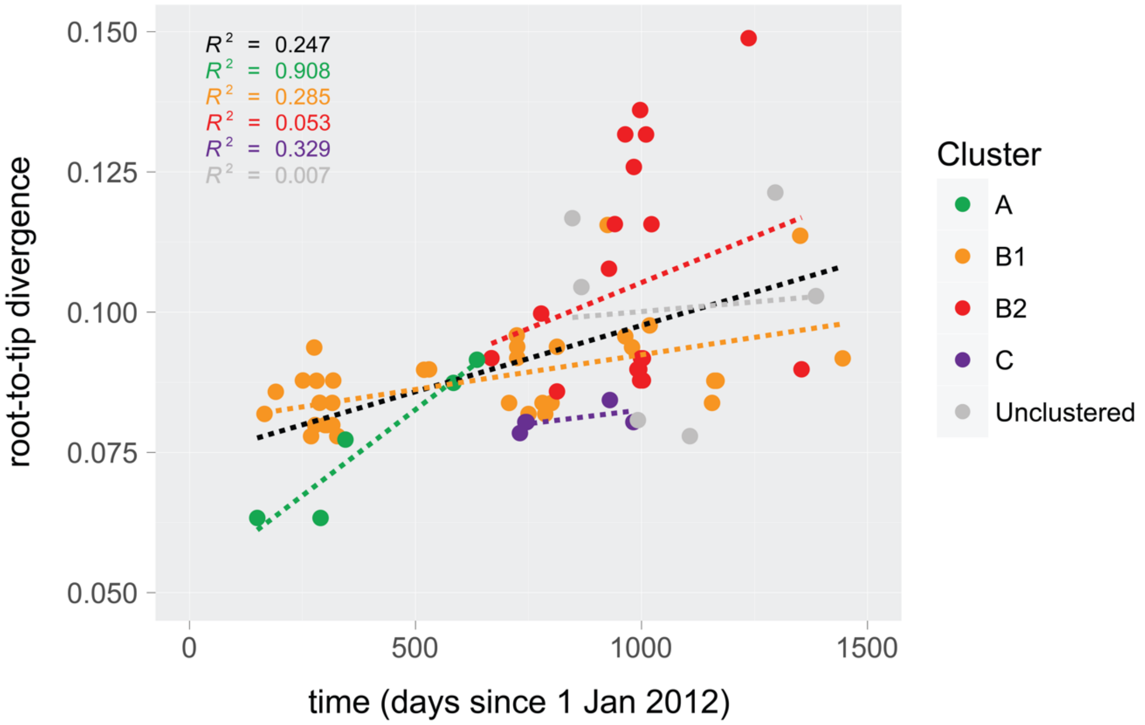

Figure S7: Temporal signal based on root-to-tip divergence. Data were generated using *TempEst* v1.5 [4] on SNPs filtered for recombination and plotted in R using ggplot2. The coloured regression lines and corresponding R^2 values indicate the signal for individual clades. Overall (black line), there was a poor temporal signal when comparing root-to-tip distance and date of isolate collection, with R^2 = 0.247. However, this may be in part due to clade-specific molecular clocks, with better correlation within individual clades.

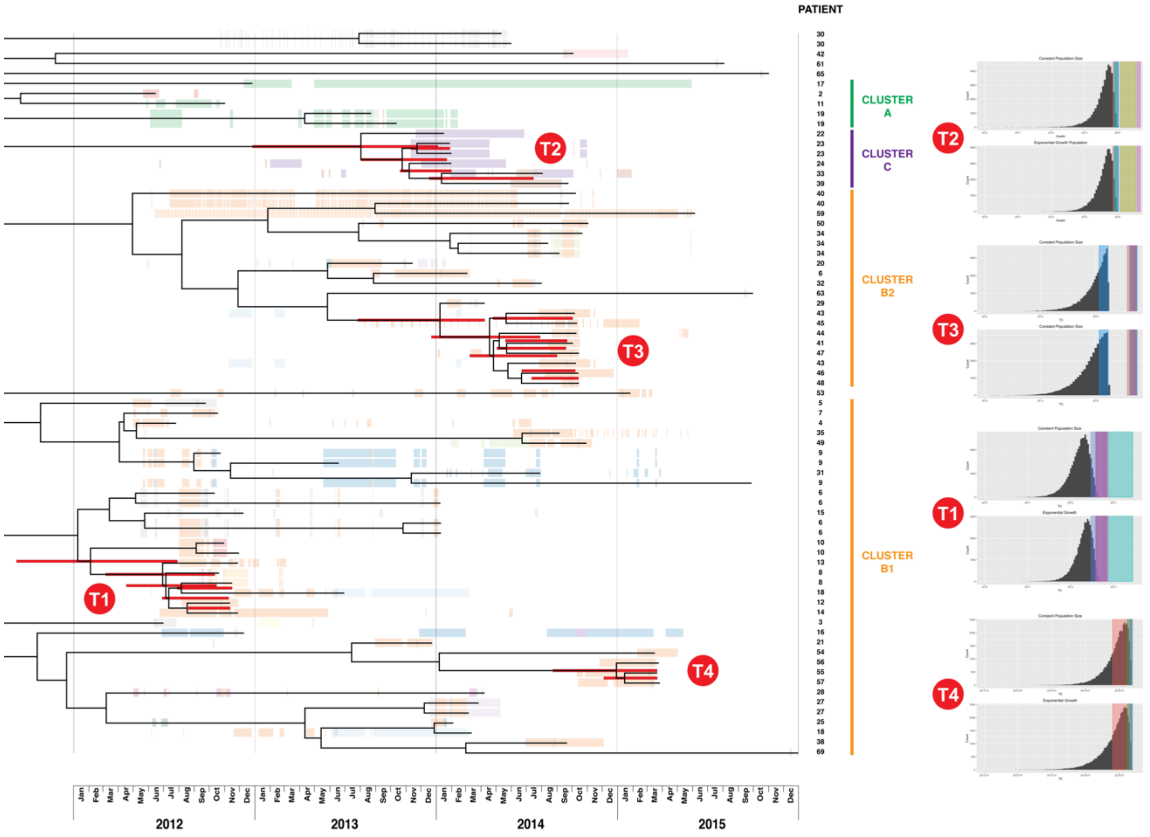

Figure S8: Posterior probability distributions for each MRCA of four primary transmission networks (T1-T4). The shaded regions behind the phylogenetic tree are coloured by healthcare facility, and indicate patient admissions over time. The corresponding distributions on the right show posterior distributions for the common ancestor nodes of each transmission network using constant and exponential population tree priors. Coloured regions indicate the period of time from hospital admission for each patient until first isolation of a KPC-producing organism. Posterior probabilities for the MRCAs for the transmission networks, T3 and T4, fell within the period that an index patient was in hospital, whereas for T1 and T2, most of the distribution preceded the patient admissions. This suggested the index isolate for each of those networks was possibly acquired by a patient prior to the documented admission (for example, the recent overseas hospitalisations of patients 22 and 23 in T2 – see Figure 6), or was derived from an unsampled individual or environmental source.

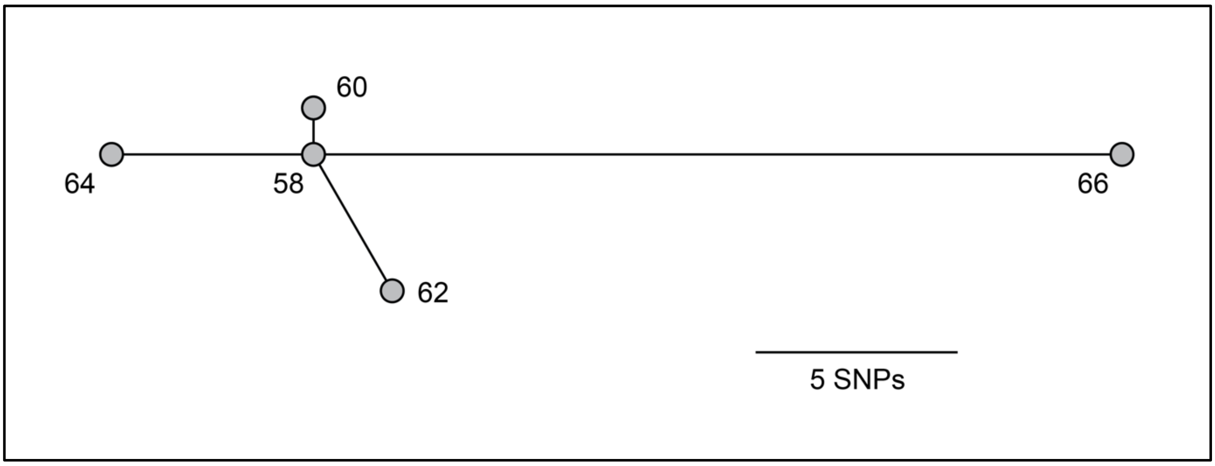

Figure S9: Neighbour-joining phylogeny of the five *Citrobacter farmeri* isolates, inferred from core genome SNPs in chromosomal sites. Nodes are labelled by patient. SNPs were called from mapping sequencing reads for each isolate against a PacBio sequenced genome from patient 60. The core genome comprised 97% of the total sites in the reference genome. No recombinant regions were detected.

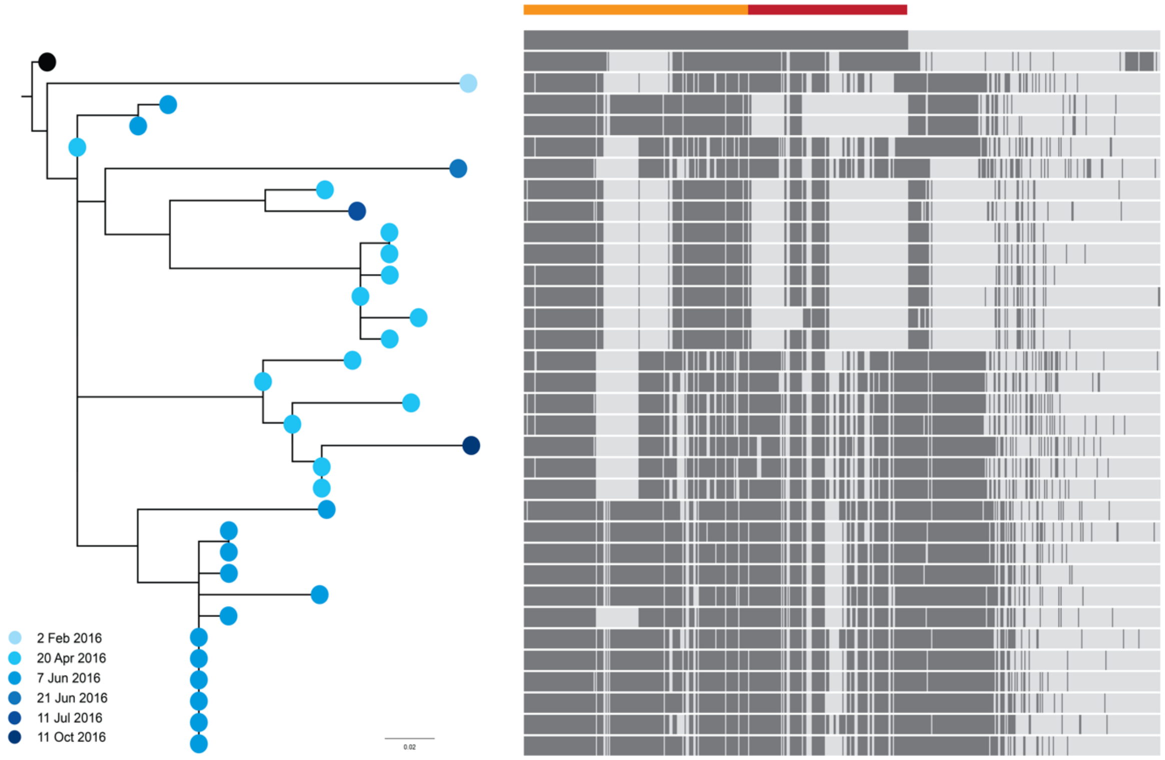

Figure S10: Maximum likelihood phylogeny and accessory genome comparison of the 32 isolates from patient 70 (nodes coloured in shades of blue, as in Figure 10), in comparison to two isolates from the suspected transmission source (black). The accessory genome is displayed on the right as a heatmap, with each row corresponding to the isolate represented in the phylogenetic tree on the left. Gene presence in the heatmap is shown as a vertical dark grey line, with light grey regions representing gene absence. Genes in the heatmap have been ordered in accordance with two plasmids (orange and red bars) in one of the isolates from the suspected source.

Table S6: Factors contributing to KPC transmission and measures undertaken to control ongoing transmission

<table>
<tr><th>Factors identified by infection control staff contributing to KPC transmission</th><th>Bundled measures undertaken in every instance where ongoing transmission was halted</th></tr>
<tr><td rowspan="3">Missed identification of patient contacts e.g. only room contacts screened, with subsequent transmission to other contacts on the same ward
Absence of permanent patient alerts for discharged patient contacts, or colonised patients
Environmental contamination of patient bathrooms
Shared bathrooms for colonised patients, due to the unavailability of single bathroom facilities
Lack of a central colonised patient registry, and inability of healthcare facilities to identify KPC colonised patients identified at other facilities</td><td>Use of contact screening
Enhanced cleaning
Isolation of cases in contact precautions requiring staff use of personal protective equipment (gloves and gown) and promotion of hand hygiene</td></tr>
<tr><td>Bundled measures undertaken in some instances where ongoing transmission was halted</td></tr>
<tr><td>Staff cohorting
Screening of patient transfers
Notification of KPC cases and contacts to receiving facilities
Environmental screening & decontamination</td></tr>
</table>

References

1. Tumbarello M, Trecarichi EM, Tumietto F, Del Bono V, De Rosa FG, Bassetti M, et al. Predictive models for identification of hospitalized patients harboring KPC-producing Klebsiella pneumoniae. Antimicrob Agents Chemother. 2014;58(6):3514-20. doi: 10.1128/AAC.02373-13. PubMed PMID: 24733460; PubMed Central PMCID: PMC4068482.
2. Price MN, Dehal PS, Arkin AP. FastTree 2--approximately maximum-likelihood trees for large alignments. PLoS One. 2010;5(3):e9490. doi: 10.1371/journal.pone.0009490. PubMed PMID: 20224823; PubMed Central PMCID: PMC2835736.
3. Cheng L, Connor TR, Siren J, Aanensen DM, Corander J. Hierarchical and spatially explicit clustering of DNA sequences with BAPS software. Mol Biol Evol. 2013;30(5):1224-8. doi: 10.1093/molbev/mst028. PubMed PMID: 23408797; PubMed Central PMCID: PMC3670731.
4. Rambaut A, Lam TT, Max Carvalho L, Pybus OG. Exploring the temporal structure of heterochronous sequences using TempEst (formerly Path-O-Gen). Virus Evol. 2016;2(1):vew007. doi: 10.1093/ve/vew007. PubMed PMID: 27774300; PubMed Central PMCID: PMC4989882.
5. Endimiani A, Carias LL, Hujer AM, Bethel CR, Hujer KM, Perez F, et al. Presence of plasmid-mediated quinolone resistance in Klebsiella pneumoniae isolates possessing blaKPC in the United States. Antimicrob Agents Chemother. 2008;52(7):2680-2. doi: 10.1128/AAC.00158-08. PubMed PMID: 18426899; PubMed Central PMCID: PMC2443894.
6. Poirel L, Heritier C, Tolun V, Nordmann P. Emergence of oxacillinase-mediated resistance to imipenem in Klebsiella pneumoniae. Antimicrob Agents Chemother. 2004;48(1):15-22. PubMed PMID: 14693513; PubMed Central PMCID: PMC310167.
7. Pitout JD, Gregson DB, Poirel L, McClure JA, Le P, Church DL. Detection of Pseudomonas aeruginosa producing metallo-beta-lactamases in a large centralized laboratory. J Clin Microbiol. 2005;43(7):3129-35. doi: 10.1128/JCM.43.7.3129-3135.2005. PubMed PMID: 16000424; PubMed Central PMCID: PMC1169086.
8. Mushtaq S, Irfan S, Sarma JB, Doumith M, Pike R, Pitout J, et al. Phylogenetic diversity of Escherichia coli strains producing NDM-type carbapenemases. J Antimicrob Chemother. 2011;66(9):2002-5. doi: 10.1093/jac/dkr226. PubMed PMID: 21669947.
9. Yan JJ, Hsueh PR, Ko WC, Luh KT, Tsai SH, Wu HM, et al. Metallo-beta-lactamases in clinical Pseudomonas isolates in Taiwan and identification of VIM-3, a novel variant of the VIM-2 enzyme. Antimicrob Agents Chemother. 2001;45(8):2224-8. doi: 10.1128/AAC.45.8.2224-2228.2001. PubMed PMID: 11451678; PubMed Central PMCID: PMC90635.
10. Hornsey M, Phee L, Wareham DW. A novel variant, NDM-5, of the New Delhi metallo-beta-lactamase in a multidrug-resistant Escherichia coli ST648 isolate recovered from a patient in the

United Kingdom. Antimicrob Agents Chemother. 2011;55(12):5952-4. doi: 10.1128/AAC.05108-11. PubMed PMID: 21930874; PubMed Central PMCID: PMC3232805.

11. Perez-Perez FJ, Hanson ND. Detection of plasmid-mediated AmpC beta-lactamase genes in clinical isolates by using multiplex PCR. J Clin Microbiol. 2002;40(6):2153-62. PubMed PMID: 12037080; PubMed Central PMCID: PMC130804.
12. Woodford N, Ellington MJ, Coelho JM, Turton JF, Ward ME, Brown S, et al. Multiplex PCR for genes encoding prevalent OXA carbapenemases in Acinetobacter spp. Int J Antimicrob Agents. 2006;27(4):351-3. doi: 10.1016/j.ijantimicag.2006.01.004. PubMed PMID: 16564159.
13. Turton JF, Ward ME, Woodford N, Kaufmann ME, Pike R, Livermore DM, et al. The role of ISAba1 in expression of OXA carbapenemase genes in Acinetobacter baumannii. FEMS Microbiol Lett. 2006;258(1):72-7. doi: 10.1111/j.1574-6968.2006.00195.x. PubMed PMID: 16630258.
